# Thyme Antimicrobial Effect in Edible Films with High Pressure Thermally Treated Whey Protein Concentrate

**DOI:** 10.3390/foods9070855

**Published:** 2020-06-30

**Authors:** Iulia Bleoancă, Elena Enachi, Daniela Borda

**Affiliations:** Faculty of Food Science and Engineering, Dunarea de Jos University of Galati, 800201 Galati, Romania; Iulia.Bleoanca@ugal.ro (I.B.); Elena.Ionita@ugal.ro (E.E.)

**Keywords:** thyme, essential oil, edible films, high pressure thermal treatment, ultrasonication, antimicrobial, thymol, carvacrol, food safety

## Abstract

Application of high pressure-thermal treatment (600 MPa and 70 °C, 20 min) for obtaining edible films functionalized with thyme extracts have been studied in order to evaluate the antimicrobial capacity of films structure to retain and release the bioactive compounds. The high pressure-thermally treated films (HPT) were compared with the thermally treated (TT) ones (80 ± 0.5 °C, 35 min). The film structures were analyzed and the sorption isotherms, water vapor permeability, antimicrobial activity and the volatile fingerprints by GC/MS were performed. The HPT film presented more binding sites for water chemi-sorption than TT films and displayed significantly lower WVP than TT films (*p* < 0.05). TT films displayed slightly, but significant higher, antimicrobial activity (*p* < 0.05) against *Geotrichum candidum* in the first day and against *Bacillus subtilis* in the 10th day of storage. The HPT film structure had ~1.5-fold higher capacity to retain volatiles after drying compared to TT films. From the HPT films higher amount of p-cymene and α-terpinene was volatilized during 10 days of storage at 25 °C, 50% RH while from the TT films higher amount of caryophyllene and carvacrol were released. During storage HPT films had a 2-fold lower capacity to retain monoterpenes compared to TT films.

## 1. Introduction

Consumers’ increasing demand for minimally processed food products led to increased researchers’ attention towards new ways to valorize the potential of plant-based extracts as preservatives for extending food shelf-life and insuring food safety. Essential oils (EOs), used conventionally as flavorings by the food industry are considered for new applications as antimicrobials and antioxidants and are generally recognized as safe (GRAS) by the United States Food and Drug Administration [1]. In the EU, Regulation 1334/2008 sets the maximum levels of certain substances present as flavorings in or on foods, EOs included. 

The biological properties of EOs are determined by its components, which are typically low molecular weight terpenes and terpenoids, nonetheless other aromatic and aliphatic molecules could be present. From the aromatic plants volatile profile, terpenes (C10) are representing 90% of the EOs but sesquiterpenes (C15) are also frequently present [2]. Even though EOs have antimicrobial effect against a wide range of food related spoilage and pathogenic microorganisms, the required concentration is often too high and their intense odor may negatively interfere with food quality and consumers’ acceptance. One solution to reduce the negative effect of EOs on food flavor is the inclusion of EOs into edible packaging, such as films and coatings.

Edible films (EF) are thin layers of edible materials (polysaccharides, proteins and lipids, and the combination of two or more of the above), which once formed can be placed on or between food components [3].

EF functionalized with EOs act as antimicrobial and antioxidant carriers, enabling their release at the interface between packaging and food product while maintaining the antimicrobial effect [4] and preserving food quality for longer periods of time.

Recently it has been demonstrated that lemongrass EOs have succeeded to limit the extent of depolymerization in chia mucilage emulsion and prevented autooxidation [5]. To overcome the EOs inherent photo-, thermal-sensitivity coupled with their high volatility micro and nanoencapsulation methods have been employed [6,7,8,9].

In the same time, edible packagings are an environment- friendly solution as their constituents are fully biodegradable and in some cases they valorize industrial waste, as is the case of whey protein recovered from the cheese- making process. Nonetheless, EOs incorporation into EF change their most relevant properties, such as the continuity of polymer matrix, weakening the film structure, reducing its transparency, while improving water barrier properties [3]. In this regard it is necessary to investigate the specific interactions between the polymer matrix and the EOs composition in order to determine the effectiveness of EOs as active ingredients. Application of whey proteins with EOs in EF has been investigated by several researchers showing the EOs’ antimicrobial effect, the excellent oxygen barrier properties, transparency but the relatively low water vapor permeability of the films [10,11,12]. To favor film formations, the whey proteins should undergo thermal denaturation, above 70 °C. Further, the unfolded globular whey proteins, expose the buried SH groups and hydrophobic groups that can react forming inter- and intra-molecular bonding during film drying [13]. Besides thermal treatment, ultrasound and addition of transglutaminase have been tested for protein denaturation prior EF drying [13]. These alternative methods could also dictate the capacity of film structure to retain and gradually release the volatiles compounds, but also influence the mechanical properties and water vapor permeability of films.

High pressure processing (HPP) is an alternative to thermal treatment that can induce structural changes in macromolecules which are distinct from those of conventional thermal treatment [14]. However, to favor the protein film formation, a combination of high pressure with thermal treatment is required. Due to the different mechanism involved in protein denaturation, high pressure thermal processing (HPT) could result in the formation of a protein-based network with different properties compared to thermal treatment (TT).

In this study, combined high pressure at 600 MPa with thermal treatment (70 °C) was employed as original alternative to thermal treatment alone for whey protein aggregation, promoting intermolecular interactions between film forming substances, which are crucial for film forming step [15]. For obtaining a homogenous film forming emulsion, ultrasound treatment was used here [16].

The objective of this research was to obtain a homogenous, flexible, resistant film formulae made of whey proteins and functionalized with thyme EO (TEO) as antimicrobial agent. The films obtained by casting were further characterized to assess their potential for food packaging applications, in terms of mechanical, physico-chemical and antimicrobial properties. The capacity of HPT and TT films to retain and release the EOs trapped in the films structure was assessed over time in relation with their antimicrobial activity.

## 2. Materials and Methods

### 2.1. Materials

Whey protein concentrate, ProMilk 852FB1 was kindly offered by KUK-Romania (composition on dry-weight basis: 86% protein, 1% total fat, 11% lactose, 2.9% total ash, 5% moisture). Anhydrous glycerol (98% purity) purchased from Redox SRL (Bucharest, Romania). Tween 20, was purchased from Sigma- Aldrich (Bucharest, Romania). Thyme (*Thymus vulgaris*) EO, kindly provided by SC Hofigal SRL (Bucharest, Romania).

### 2.2. Film Preparation 

The film was prepared by dispersing 7.6% (*w*/*w*) WPC powder, into distilled water under continuous magnetic stirring (180 rpm, 15 min) following a method previously optimized by Bleoanca et al. [17]. The pH was adjusted to 7.0 using 2 N NaOH [18]. In order to transform the protein solution in a flexible film either thermal crosslinking (80 ± 0.5 °C, for 35 min) or combined HPT denaturation (600 MPa, 70 °C, for 20 min) were applied.

#### 2.2.1. Thermal Treatment

The thermal inactivation was applied in a thermostatic water bath at 80 ± 0.5 °C for 35 min. Timing was started after the temperature measured inside the sample has reached 80 °C, as measured by type K thermocouple in one of the glass vials. Immediately after finishing the thermal treatment the samples were cooled in iced-water to stop the thermal effect.

#### 2.2.2. Combined Mild-Thermal High Pressure Treatment

Combined pressure- temperature treatments were conducted in a multivessel (4 vessels of 100 mL) high-pressure equipment (Resato, Roden, The Netherlands). As a pressure transmitting fluid, a mixture of water and propylene glycol (TR15, Resato) was used. The sample, approximately 30 mL, was first heated at 65 °C, and then filled without air into Teflon cylinders and placed into the HPP vessels to avoid temperature gradients. Compression started when the temperature was equal to target temperature, 70 °C, up to 600 MPa, and 20 min holding times. The compression rate was of approximately 10 MPa/s, until the preset pressure was reached, whereupon the valves of the individual vessels were closed and the central circuit was decompressed. An additional one-minute equilibration period was taken into account to ensure constant temperatures. Temperature inside the samples was monitored during the treatment with a thermocouple placed in the upper part of the Teflon cylinders. Decompression of the vessels was almost instantaneously (~5 s). After the pressure-temperature treatment, the samples were immediately transferred into iced water.

After forced cooling on ice, into the resulting film forming mixture obtained either by thermal or combined high pressure- thermal denaturation, anhydrous glycerol was added at a concentration of 8.0% (*w*/*w*) as plasticizer to reduce the brittleness of the WPC films, thus improving its mechanical properties. As surfactant for reducing the surface tension, tween 20 was used in a concentration of 0.9% (*w*/*w*). Then, thyme (*Thymus vulgaris*) EO, was added in the mixture as antimicrobial compound in a concentration of 2.5% (*w*/*w*). This plant EO was chosen due to its high content of carvacrol, thymol and p-cymene, all known to be efficient antimicrobials [19] and considering the results of previous tests performed by our research group [20].

Further the mix was homogenized by ultrasonication with equipment Sonoplus HD3100 Bandelin, Germany equipped with a sonication probe of 8 mm diameter, at 35% amplitudes, for 3 min. The sonication probe was immersed 1 cm below the liquid surface and the temperature of the film forming emulsion was kept at 23 ± 2 °C during sonication by placing the tube in an iced water bath [16].

The film forming emulsion was then poured onto silicone trays (diameter 5 cm). To control film thickness, the same amount (11 mL) of film forming mixture was poured. The spread solutions were allowed to dry at room temperature, approximately 22 °C, for 48 h at 50% RH [21,22], then easily peeled off.

Considering the hydrophilic nature of the protein film, therefore its susceptibility to absorb humidity from the environment, a standardization of the films was necessary to ensure that the mechanical properties of the film are not impaired. For this reason, prior to all investigations, the films were preconditioned by storing them in a controlled temperature- humidity environment, at 50 ± 3% RH and 25 ± 1 °C, for at least 72 h [23].All the experiments were performed in triplicate.

### 2.3. Film Characterization

#### 2.3.1. Film Thickness

A digital micrometer (Digimatic Micrometer, Mitutoyo, Japan) was used to measure film thickness to the nearest 0.0001 mm. The mean thickness was calculated from five measurements taken randomly at different locations on each film.

#### 2.3.2. Moisture Content

The moisture content (MC) of the whey protein films was determined after oven drying at 105 ± 1 °C for 24 h until a constant weight was attained. After adequate conditioning, 3.4 cm diameter discs were cut from the edible film and weighed in order to be compared to the ones after drying. The moisture content values were determined as percentage of initial film weight loss during drying [24].

(1) $$\mathrm{MC}=\frac{w_{1}-w_{2}}{w_{1}-w_{0}}\times 100\;[\%]$$

*w*_0_ is the weight of empty and dry weighing glass bottle, (g); *w*_1_ is the weight of weighing glass bottle with film, before drying, (g); *w*_2_ is the weight of weighing glass bottle with film, after drying, (g).

#### 2.3.3. Water Activity

The water activity (*a*_w_) of preconditioned edible films was measured with a (Fast lab water activity meter; GBX, Loire, France), using discs of films (4 ± 0.1 cm diameter).

#### 2.3.4. Moisture Sorption Isotherms

Moisture sorption isotherms were determined by static gravimetric method [25]. Dried film samples were first conditioned for 5–10 days into a controlled humidity environment at a constant temperature until equilibrium has been reached. Samples discs of 49.58 ± 0.31 mm were placed into desiccators, each containing one saturated salt solution giving various RH at 25 °C: LiCl for an a_w_ of 0.114, MgCl_2_ giving a 0.331 *a*_w_, KI giving an *a*_w_ of 0.700, NaCl for an *a*_w_ of 0.755, KCl giving an *a*_w_ of 0.851 and KNO_3_ for an *a*_w_ of 0.935. Film samples were equilibrated at each environment for 5–10 days at 25 ± 0.5 °C; following removal from desiccators they were immediately weighed, the *a*_w_ was determined and moisture content was measured gravimetrically as described above. The Guggenheim-Anderson-de-Boer and Halsey models [26] as indicated by Tudose et al. [27] were applied by nonlinear regression analysis (SAS, 2009):

(2) $$M=\frac{M_{o\;}\times C\times K\times a_{w}}{\left(1-K\times a_{w}\right)\times \left(1-K\times a_{w}+C\times K\times a_{w}\right)}$$

where *M* is the equilibrium moisture content (% dry basis); *M*_0_ is the monolayer moisture content (% dry basis); *C*—Guggenheim constant; *K*—corrective constant; *a*_w_ is the water activity (dimensionless);

The Halsey equation is:

(3) $$a_{w}=\exp\left(-\frac{k}{M^{n}}\right)$$

where *k* and *n* are model constants.

#### 2.3.5. Water Vapor Permeability

Water vapor permeability (WVP) was estimated gravimetrically according to ASTM E96 [28], adapted for edible films. Film discs of 49.58 ± 0.31 mm diameter equilibrated at 25 °C, 50% RH for 48 h with saturate salt solution (Mg(NO_3_)_2_) were cut and mounted on glass cups filled with distilled water to 10 mm below the film underside. The glass cups had 46 mm diameter and 150 mm depth. The steady-state films water- vapor flow was measured at certain intervals for 48 h by digital-balance nearest to 0.0001 g. Films permeability was calculated according to the method described by Zinoviadou et al. [11]. The weight loss was monitored and expressed by the slopes calculated using linear regressions equations where *R*^2^ > 0.99. At least five replicates were tested for WVP estimation.

(4) $$WVP=\frac{Slope\times x}{A\times \Delta p}\;(g\text{·}\mathrm{mm}/m^{2}\text{·}s\text{·}\mathrm{Pa})\;$$

where *slope* is the weight loss of the cup per second, (g/s); *x* is the average film thickness, (mm); *A* is the area of exposed film, (m^2^); Δ*p* is the difference in vapor pressure across the test film (Pa).

#### 2.3.6. Microstructural Analysis of The Film Forming Mixtures

A scanning electron microscope (Quanta 250, Thermo Fisher Scientific) (Waltham, MA 02451, USA) was used to determine the microstructure of thermal treated (TT) and combination of high pressure with temperature treatment (HPT) whey protein film samples with an accelerating voltage of 12.5 kV in a low vacuum environment. A magnification of 400×–1400× was used to scan each film sample.

### 2.4. Antimicrobial Assay

The antimicrobial effect of edible films was tested against three target microorganisms, *Bacillus subtilis*, *Geotrichum candidum* and *Torulopsis stellata,* all part of MIUG collection from Dunarea de Jos University of Galati- Romania. The antimicrobial effectiveness of the edible films was tested 10 days after the films were obtained, by vapor phase test [29]. This specific indirect contact assay for testing the antimicrobial activities was chosen to assess the protection provided by the thyme antimicrobial volatiles under no direct contact between the food product and the packaging. To perform vapor-phase diffusion tests, edible films of approx. 50 mm diameter discs were placed on the lids of Petri dishes, with previously spread 10^6^ cfu/mL microbial inoculum. The inoculated agar plate was inverted with discs on the top of each lid containing antimicrobial film. Parafilm was used to tightly seal the edge of each Petri dish. Sealed and inverted Petri dishes were incubated at 27 °C for evaluation of anti- *Torulopsis* and anti- *Geotrichum* activity and at 37 °C for anti-*Bacillus* activity. Growth of each test microorganism was evaluated after two days of incubation. The inhibition radius (absence of growth) on each Petri dish was measured with a digital caliper and the inhibition area was calculated and expressed as mm^2^. The negative control, represented by whey protein EF without TEO, were also tested under the same conditions. The vapor phase inhibition test was performed in duplicate, in two separate experimental runs.

### 2.5. Solid-Phase Micro-Extraction (SPME)

Before analysis the HPT and TT films were placed in desiccators of 6 L capacity with Mg(NO_3_)_2_ salt at 25 °C and 50% RH and stored for maximum 10 days. Each film had a 19.65 cm^2^ surface exposed to the environment and 3 discs were present in each desiccator for all the duration of the experiments. From each film discs with 34 mm diameter were cut, weight, introduced in sealed vials and maintained at 40 °C for 10 min for equilibration before concentration by SPME on a CAR/PDMS fiber. The extraction of the volatiles under isothermal conditions at 40 °C was made over 30 min followed by 5 min of desorption into the GC injection port.

### 2.6. Gas Chromatography-Mass Spectrometric (GC-MS) Analysis

The volatiles fingerprints of the edible film samples were analyzed using a Trace GC-MS Ultra equipment with ionic trap- ITQ 900 from Thermo Scientific (USA). The GC column was a TG-WAX capillary column (60 m × 0.25 mm, i.d. 0.25 μm). The carrier gas was helium (99.996% purity, Messer S.A., Bucharest, Romania) that ran at a flow rate of 1 mL/min. The temperature ramp selected for the analysis was: 40 °C isothermal treatment for 4 min followed by an increase to 50 °C at 5 °C/min and to 100 °C with 7 °C/min, to 150 °C at 10 °C/min and finally to 230 °C at 12 °C/min, when temperature was kept constant for 2 min. The temperature of the transfer line in MS was set to 270 °C. Mass spectra were obtained from the full scan of the positive ions resulted with a scanning in the 35 to 450 *m*/*z* range and operated with an electron impact (EI)-mode of 200 eV. The compounds were identified in comparison with the mass spectra from Wiley and Nist 08 library database available with Xcalibur 2.1 software. The retention indices (RI) of each compound were calculated by using n-alkane series from C8-C40 (Sigma Aldrich Chemie GmbH, Steinheim, Germany) under the same conditions. Each analysis was performed in triplicate, in the first and the 10th day of storage.

The volatile organic compounds (VOCs) were estimated semi-quantitatively using n-octanol as internal standard (IS) and Equation (5) [20,30]:

(5) VOCconc=ISconc×(VOCpeak areaISpeak area)

where *VOC_peak area_* is the area of the integrated individual peak, *IS_peak_*_area_ is the area of 2-octanol in the spiked samples and *IS_conc_* is the concentration of internal standard (2-octanol).

### 2.7. Statistical Analysis

Data were expressed as mean ± standard deviation (SD). The statistical analysis was carried out using analysis of variance (ANOVA) and Tuckey’ s post-hoc test was applied to evaluate significant differences among groups (*p* < 0.05).

The quality of the sorption isotherms models’ fit applied was evaluated by the regression coefficient (*R*^2^_adj_) and the mean relative percentage deviation (%*E*):

(6) $$E=\frac{100}{N}\overset{N}{\underset{i=1}{\sum }}\frac{\left|m_{i}-m_{pi}\right|}{m_{i}}$$

where *m*_i_ and *m*_pi_ are the experimental and predicted values, respectively, and *N* is the population of the experimental data.

(7) $$R_{adj}^{2}=1-\left(\frac{n_{t}-1}{n_{t}-n_{p}}\right)\cdot \frac{SSE}{SSTO}$$

Principal component analysis (PCA) was performed using the Unscrambler software (Version 9.7; CAMO, Norway). PCA was performed with the peak list resulting from SPME GC/MS analysis for all the volatile compounds. The data matrix was formed by *n* = 6 cases and 25 variables defined as the VOCs peak areas obtained for each individual component. Data were transformed by unit vector normalization prior to statistical analysis.

## 3. Results and Discussion

### 3.1. Film Appearance

Appearance of the two sides of the WPC film was similar for HPT and TT films. The film side facing the casting plate was shiny, while the other was dull; this is likely an indication of some phase separation occurring in the mixture during drying. HPT and TT types of film were easily separated from the casting plates. During the TT the three dimensional structure of proteins was unfolded and the internal sulfhydrilic groups were exposed, later forming intermolecular disulfide bonds while hydrophobic groups interactions also might have occurred during film drying [18,31]. Combination of HPP and TT resulted in both denaturation via above referred mechanism and by forcing the water molecules inside the protein matrix, that exposed the hydrophobic core, followed by protein unfolding [32,33].

Films manufactured from WPC with 7.6%(*w*/*w*) protein showed a thickness of 0.133–0.193 mm, close to those reported by other researchers [34,35,36]. Neither one of the HPT and TT WPC-based films functionalized with thyme essential oils (TEO) did not exhibit any statistically significant differences either (*p* < 0.05) (Table 1).

### 3.2. Sorption Isotherms

Sorption isotherms were studied at 25 °C and equilibrium moisture content. The data obtained confirmed the distribution on a sigmoidal shaped curve, characteristic of type II isotherms observed for most of the biopolymer materials and foods.

Table 2 presents the GAB and Halsey model parameters estimated for the two films formulation TT and HPT. The values indicating the goodness of the model fit to the experimental data are showing that both GAB and Halsey models are adequate for the data with %E 0.197–1.21 and a good agreement between experimental and predicted data (*R*^2^_adj_ = 0.89 ÷ 0.99).

However, the *R*^2^_adj_ and E-values for GAB have better values than for Halsey. GAB has the advantage of providing information on the monolayer water content (M_0_) that indicates the number of the sorbing sites and the maximum amount of water that can be absorbed [37,38]. The values indicated by the current study are close to the ones reported by Wang et al. [39] who demonstrated that WPC films are able to adsorb more moisture than casein films. Similar values were also registered by Silva et al. [40] and Huntrakul and Harnkarnsujarit [37] and lower values were recorded by Zinoviadou et al. [11] for the whey protein isolate films with oregano compared to this study.

An almost twice higher value was obtained for the HPT film compared to TT thus it can be presumed that HPT films had more binding sites for water chemi-sorption than TT films and this could make more susceptible to swelling.

### 3.3. Water Vapor Permeability (WPV)

The water vapor permeability (WVP) and the film thickness are presented in Table 2. WVP of food packaging is an important parameter that gives information on sorption, diffusion and adsorption. Low values of WVP are desired for the edible films since one of the required characteristics of the edible film is to retard moisture transfer between the food product and the environment [41]. The WVP of the films with TEO treated by HPP have a significant lower permeability compared to the TT films. The values reported in this study for the 46–100% RH are in the same range, however slightly lower than the ones with those reported by Kokoszka et al. [34] for whey protein isolates and by [42] for WPC. However, compared to Kokoszka et al. [34] in our case WPC, tween and TEO was added in film formulation. The WPV was lower than the values reported by Bahram et al. [42], but the amount of essential oil used in this case was higher (2.5%) than the maximum amount used in the films with cinnamon oil (1.5%). Compared to control (control TT and control HPT) the films with TEO added (HPT and TT) had a significantly (*p* < 0.05) lower WVP (Table 2), explained by the increase in hydrophobicity and observed also by other researchers when EOs were added to the film structure [42,43].

### 3.4. Scanning Electron Microscopy

**Figure 1:**
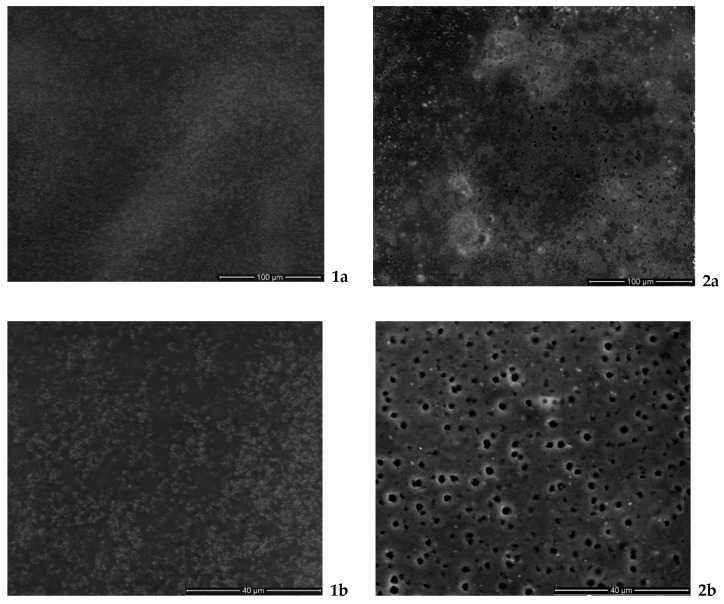
Surface morphology of TEO WPC EF. The film forming mixture was denatured either by TT (**1**) or by HPT (**2**). Surfaces viewed at magnification of 400× (**a**) and 1400× (**b**). TEO: thyme EO; EF: Edible films ; TT: thermal treated; HPT: high pressure-thermally treated.

Figure 1 illustrates the SEM micrographs of TT (1) and HPT (2) TEO WPC films surface. The microstructure of the films reveals the structural arrangement of its components that influence both physical and mechanical properties of the films [44].Microscopy images of edible films surface show continuous, compact and homogenous structures, without any irregularities such as air bubbles or cracks. Nonetheless, the TT films are more homogenous and exhibit a smoother film surface compared to the HPT ones, that could be due to different intermolecular interactions mechanisms. At a higher magnification, the TEO droplets can be easily observed in the HPT films compared to the TT films. Moreover, the TEO droplets are scarcely observable in the TT samples, which could be related to their better integration in the thermal denatured whey protein matrix compared to the case of HPT protein denaturation.

Previous researches have shown that film microstructure is also correlated with mechanical and optical properties of the EFs [44]; however this properties were not investigated by the current study.

### 3.5. Antimicrobial Effect of PFunctionalizing the WPC-EF

The antimicrobial activity of EOs has been intensively studied and is well recognized. The growing published evidence towards a more effective antimicrobial activity of EOs in vapor phase compared to EOs in liquid form applied by direct contact [45,46,47] led to identification of new applications for EOs vapors, including those in the food industry [46,47]. One plausible explanation for the different antimicrobial effectiveness is the mechanism presented by the group of researchers Nadjib et al. [48] indicating formation of micelles from association of lipophilic molecules in the aqueous phase which negatively interfere with the EOs attachment to the microorganisms, while the EO vapors allow free attachment to microorganism’s cells.

The current study evaluated the antimicrobial effect by vapor phase diffusion method of TEO functionalizing the WPC-EF against three test microorganisms. The current TEO WPC-EF is intended to function as an active antimicrobial food packaging providing microbial surface protection of the fresh food product by effectively controlling the growth of aerobic microorganisms through the volatile antimicrobials released into the food package headspace.

**Figure 2:**
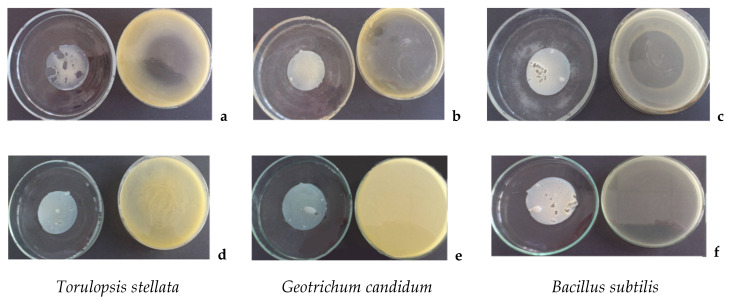
Sample pictures of vapor phase test (**a**–**c**) of TEO WPC- EF on test microorganisms *Torulopsis stellata*, *Geotrichum candidum* and *Bacillus subtilis*. **d**–**f** are control WPC-EF without TEO.

Due to the absence of direct contact between the test microorganisms and TEO WPC-EF, this method allowed the detection of the antimicrobial potency of volatile components exclusively. Results of the antimicrobial activity of 2.5% (*w*/*w*) TEO WPC-EF through vapor phase test are presented in Figure 2 and Table 3.

In vitro assessment of sensitivity to thyme volatiles of three spoilage test microorganisms of environmental origin was evaluated by vapor phase assay. TEO functionalizing both types of EFs, TT and HPT, showed effective antimicrobial activity based on the inhibition zones against all three fresh products spoilage microorganisms. For *Torulopsis stellata* inhibition zones ranged between 9.00 to 17.50 mm, with no significant statistical differences between TT and HPT EFs during the 10 days tested. *Geotrichum candidum* produced inhibition halos higher than *Torulopsis stellata,* up to 20.00 mm after 10 days for HPT-EF. Significant differences in terms of thyme antimicrobial efficacy against *Geotrichum candidum* were observed only for the first day of test, higher for TT-EFs. *Bacillus subtilis* proved to be the most sensitive of all three tested microorganisms, with inhibition halos ranging from 15.50 to 39.00 mm.

When comparing protein denaturation treatments, TT with HPT, the antimicrobial activity of the HPT- EF against *Torulopsis stellata* after 10 days of storage, no significantly differences (*p* > 0.05) compared to the other samples, with higher inhibition radius for TT-EF. Thyme antimicrobial effect against *Geotrichum candidum* is significantly higher in TT-EF in the beginning, on day 1 compared to day 10, however no significant differences was registered after 10 days of storage between the TT and HPT films. For *Bacillus subtilis* the antimicrobial efficacy has no significant differences (*p* > 0.05) in the first day between TT and HPT films, however throughout the 10 days evaluation the TT films displayed a slightly higher antimicrobial effectiveness (*p* < 0.05) compare to the HPT films.

Two main characteristics greatly influence the volatility of EOs components in general, here thyme in particular: one is the molecular weight of their constituents; each chemical compounds from the mixture forming EOs has a different volatility according to its molecular weight, which influences their diffusion rate when EO is introduced in a non-saturated environment, as is the case with the sealed Petri dishes used for the this diffusion assay. The other TEO characteristic is related to the denaturation treatment of proteins from film forming mixture which influences the entrapment of the EOs in the WPC matrix, as well as promoting the release of TEO out of the proteic matrix.

It is fully understood that the antimicrobial activity of the essential oils in vapor phase is closely related to its composition in the headspace [49]. However, it should be mentioned that in the case of antimicrobial activity, an additive day-by-day effect of the VOCs was evaluated on the tested microorganisms, produced by the gradual release of the VOCs from the film matrix during storage in a contained environment created by the Petri dishes.

### 3.6. Gas-Chromatography Fingerprint

**Figure 3:**
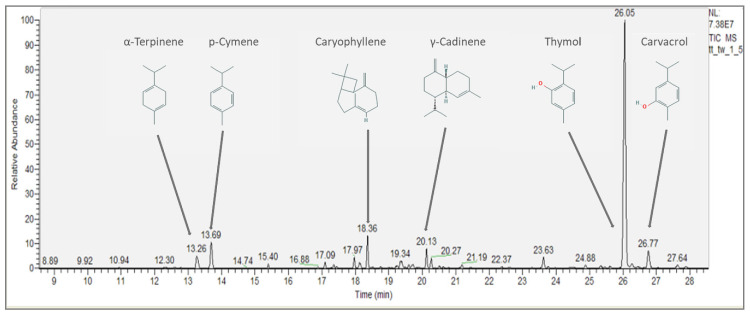
Fingerprint of the main volatiles present in the TT film functionalized with thyme, in the first day of storage.

The individual chromatograms of the tested sample are shown in the Supplementary Materials (Figures S1–S4) and the VOCs entrapped in the 2-types of matrices tested (TT, HPT) are presented in Table 4. A total number of 25 volatiles were tentatively identified using NIST library and the compounds were present in different concentrations in all the film structures analyzed where thyme has been added (Figure 3). The most abundant VOCs were the ones regularly present in TEOs [20,50], namely thymol, p-cymene, α-terpinene, and carvacrol (Table 4). Often, p-cymene and γ- terpinene are reported as precursors of thymol and carvacrol that occur in variable proportions in plants [20,51]. In this case, in the film’s matrices, only α-terpinene was identified. In all the edible films formulae p-cymene was present in high concentrations, however thymol had the highest concentrations in all films, while there were no significant differences (*p* < 0.05) in the concentrations of this compound between the two formulations (TT and HPT) (Table 4).

While in all the initially prepared emulsions the concentration of TEO added was the same, the capacity of the dried films structure to retain the VOCs can be judged as a function of the pretreatment applied. Immediately after drying, the film structure able to retain the highest concentration of the main VOCs was the HPT film that displayed in general ~1.5-fold better capacity to retain the VOCs compared to the TT film. The better capacity of HPT film to trap the VOCs compared to TT could be related to the different mechanisms involved in whey protein denaturation [14] and consequently related to the different film structure capacity to retain volatiles. High pressure treatment can be used as a tool to tailor unique properties of food structures, which may not be forthcoming through other ways of processing [14,52,53]. High-pressure predispose the whey proteins to changes in their tertiary and quaternary structures towards formation of small aggregates dominated by side-by-side interactions, enabling a narrower size distribution than thermal treatment. Usually, the changes are also associated with an increase in the apparent viscosity of the pressurized systems [54]. During HPT treatment no gelation occurred, however the samples displayed higher viscosity than the TT ones.

The combined HPT treatment resulted into a denser film compared with the thermally treated ones and with better defined individual oil droplets inside the film structure as shown by microscopy analysis (Figure 1). This observation could indicate a better entrapment capacity but a weaker linkage of TEO in HPT compared to TT films.

The dried protein films complemented with tween surfactant, glycerol and thyme that went through different preliminary processing methods (HPT and TT), were then assessed in relation with the capacity to withhold the aromatic molecules during ten days of storage. The edible films were kept at constant relative humidity (RH 50%) and environmental temperature (25 °C).

In the SPME GC-Ms analysis the samples were kept in the same equilibrium environment for 10 days and later on, they were tested, basically measuring the remaining VOCs in the edible film matrix.

**Figure 4:**
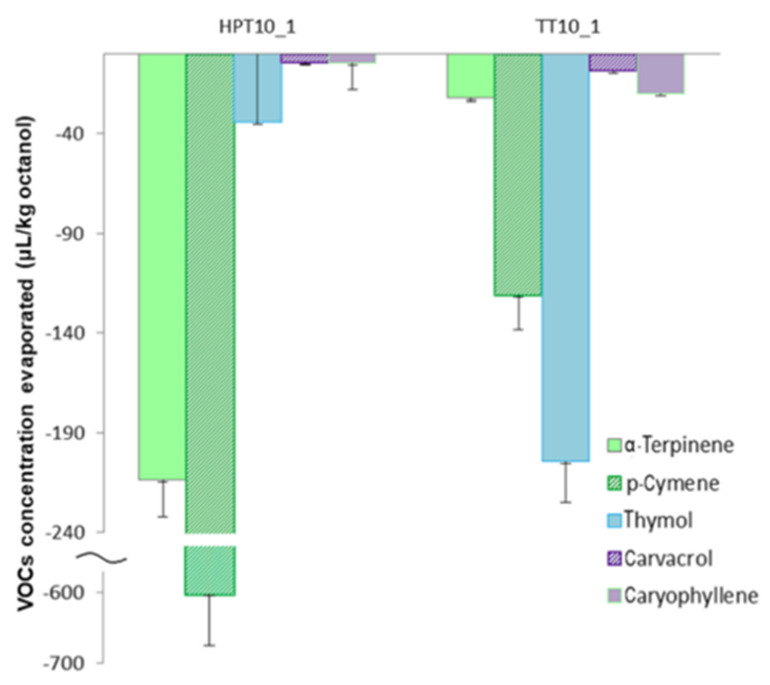
The loss of main volatiles in the HPT and TT edible films during 10 days of storage at 50 ± 3% RH and 25 ± 1 °C.

When evaluating the fingerprints of HPT and TT after 10 days it can be noticed that HPT film lost higher amounts of p-cymene (54.63%) and α-terpinene (50.06%) (HPT10 1) compared the thermally treated ones 32.03% and 25.22%, respectively (TT10_1) (Figure 4).

Another VOC that was consistently reduced by 79.90% after 10 days of storage is caryophyllene oxide in the HPT film. The most desired property of the antimicrobial packaging materials is the controlled release of the antimicrobial agents from the film to the food surface. A burst release of VOCs causes fast consumption of the antimicrobial agent after which the minimum concentration required for the inhibition of microbial growth is not maintained on the food surface [55]. On the other hand, spoilage reactions on the food surface may start if the release rate of the antimicrobial agent from the film is too slow. Thus, the controlled release of the active agent over a long period of time is necessary to extend the shelf life of the packaged food [56].

The edible film structures obtained in this research showed that HPT displayed over time a 2-fold lower capacity to retain the monoterpenes (MTs) with high volatility (KI from 935 to 1044) compared to TT. This finding demonstrates that forces involved in the VOCs entrapment in HPT treatment are weak so these components are more susceptible of fast leaving the films compared to TT. Despite the initially better capacity to retain volatiles the HPT matrix demonstrated a lower capacity to retain over storage especially the MTs with high volatility.

### 3.7. PCA Analysis

**Figure 5:**
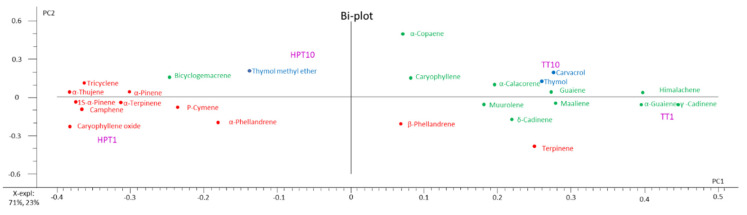
The Bi-plot of the principal component analysis of HPT and TT fingerprint during 10 days of storage at 50 ± 3% RH and 25 ± 1 °C.

The PCA could explain 94% of the total variation of VOCs in the sample with the highest contribution explained by PC1 (Figure 5). The association of the volatiles and samples given by the PCA analysis shows that the highest contribution in PC1 is made by the TT1 with α-guaiene, cadinene but also by TT10 associated with high concentrations of thymol and carvacrol. Oppositely influencing the PC1, is the HPT structure, from the first day (HPT1), containing camphene, and p-cymene. After 10 days of storage the content in bicylogemacrene and thymol methyl ether in the HPT film could explain most of the variation influencing the PC2.

## 4. Conclusions

This study showed that HPT denaturation of whey proteins result in different structures compared to the TT. The HPT films were more prone to swell and presented a lower WVP than TT films. The antimicrobial activity for the films contained in glass Petri dishes were comparable, however a slightly better antimicrobial activity of the vapors was demonstrated by the TT films against *Geotrichum candidum* in the first day and against *Bacillus subtilis* in the 10th day of storage.

The HPT functionalized with TEO film had a better capacity to embed the volatiles after drying, however over time is released more easily the monoterpenes from the film structure showing a weaker capacity to withhold the highly volatile components when compared to TT film when stored in controlled environment (25 °C, 50% RH). The use of EFs in the food industry could require either long time or short-time protection of food depending on its durability, so the selected pretreatment, either thermal of combined pressure thermal pretreatment, could be elected in relation with the type of application EFs are intended for.

The current study can be considered a starting point for future designing of EF with controlled release of thyme antimicrobial components, by understanding the molecular dynamic equilibrium between the protein matrix, TEO and environment.

## Figures and Tables

**Table 1:** Thickness and WVP of TT and HPT films ^#^.

Films	Thickness (mm)	ΔRH (%)	WPV·10^−11^ (g/s·m·Pa)
Control_TT	0.171 ± 0.163 ^a^ *	46	24.867 ± 2.855 ^a^
TT	0.193 ± 0.052 ^a^	46	19.557 ± 2.109 ^b^
Control_HPT	0.156 ± 0.043 ^a^	46	13.852 ± 1.137 ^b,c^
HPT	0.133 ± 0.071 ^a^	46	10.178 ± 1.690 ^c^

^#^ mean results ± stdev; ^*^ different letters indicate significant differences (*p* < 0.05) among columns by post-hoc Tuckey test. WVP: Water vapor permeability; TT: thermal treated; HPT: high pressure-thermally treated; RH: relative humidity.

**Table 2:** Estimated parameters of the GAB and Halsey model fit to experimental data of sorption isotherms for TT and HPT films at 25 °C.

	GAB Model			Halsey Model	
Film	K	C	M_0_	*k*	*n*
		(g water/100 g dw ^b^)			
TT	0.822 ± 0.061 ^a^	10.871 ± 0.036	12.581 ± 3.617	1582.915 ± 327.473	2.422 ± 0.195
	*R*^2^_adj_ = 0.992 E(%) = 1.212			*R*^2^_adj_ = 0.807 E(%) = 0.856	
HPT	0.692 ± 0.096	3.331 ± 0.292	23.045 ± 2.681	260.816 ± 29.281	1.925 ± 0.176
	*R*^2^_adj_ = 0.999 E(%) = 0.831			*R*^2^_adj_ = 0.891 E(%) = 0.197	

^a^ mean results ± stdev; ^b^ dw = dry weight.

**Table 3:** Inhibition and growth reduction zones provided by thyme volatiles functionalizing WPC-EF. Results are expressed in mm, as mean ± standard deviation.

	TT		HPT	
	Day 1	Day 10	Day 1	Day 10
*Torulopsis stellata*	10.50 ± 0.50 ^b,B,^*	17.50 ± 0.71 ^a,B^	9.00 ± 1.41 ^b,B^	15.00 ± 1.41 ^a,C^
*Geotrichum candidum*	16.00 ± 1.41 ^b,A^	19.50 ± 0.71 ^a,B^	10.50 ± 0.71 ^c,B^	20.00 ± 0.00 ^a,B^
*Bacillus subtilis*	16.50 ± 0.71 ^c,A^	39.00 ± 1.41 ^a,A^	15.50 ± 0.71 ^c,A^	35.00 ± 0.00 ^b,A^

* Superscripts with different letters indicate significant differences (*p* < 0.05) between the rows values (small caps) and between the column values (capital letters). by post-hoc Tuckey test; TT—Thermal treatment of film forming mixture; HPT—High pressure & thermal treatment of film forming mixture.

**Table 4:** The GC/MS SPME volatiles concentration (μL/kg octanol) in HPT and TT edible films functionalized with TEO, in the beginning of storage (HPT1, TT1) and after ten days of storage (HPT10, TT10) at constant RH and temperature (RH 50%; 25 °C).

Compound	Class	KI	Ions	HPT1	HPT10	TT1	TT10
Tricyclene	MT	935	91;93;77;121	13.96 ± 1.45 ^g,A,^*	7.92 ± 0.55 ^e,B^	2.09 ± 0.19 ^e,C^	1.69 ± 0.11 ^e,C^
α-Thujene	MT	946	91;77; 93;65	4.68 ± 0.52 ^g,A^	2.61 ± 0.18 ^e,B^	0.78 ± 0.06 ^e,C^	0.63 ± 0.04 ^e,C^
α-Pinene	MT	957	91;77;93;65	26.97 ± 2.13 ^f,g,A^	14.91 ± 1.22 ^e,B^	5.23 ± 0.44 ^d,e,C^	4.38 ± 0.36 ^e,C^
Camphene	MT	965	91;93;121;136;77	60.13 ± 5.25 ^f,g,A^	24.67 ± 2.31 ^d,e,B^	12.10 ± 1.14 ^c,d,e,C^	7.92 ± 0.80 ^e,C^
1S-α-Pinene	MT	970	91;67;79;93	58.50 ± 4.48 ^f,g,A^	29.97 ± 2.71 ^d,e,B^	10.70 ± 1.01 ^d,e,C^	9.26 ± 0.88 ^d,e,C^
α-Phellandrene	MT	974	91;93;77;139;51	18.68 ± 0.95 ^g,A^	6.62 ± 0.72 ^e,B^	8.15 ± 0.99 ^d,e,B^	3.63 ± 0.34 ^e,C^
α-Terpinene	MT	983	91;93;77;136	426.51 ± 38.74 ^c,A^	213.03 ± 20.19 ^c,B^	87.70 ± 8.99 ^c,d,e,C^	65.58 ± 6.69 ^c,d,e,C^
p-Cymene	MT	991	119;91;134;117	1125.78 ± 121.25 ^a,A^	510.79 ± 49.88 ^b,B^	377.02 ± 45.20 ^b,B,C^	256.25 ± 27.48 ^b,C^
α-Copaene	SQT	1038	105;91;119;161	11.38 ± 1.08 ^g,B^	26.44 ± 2.14 ^d,e,A^	10.82 ± 1.42 ^d,e,B^	7.73 ± 0.89 ^e,B^
β-Phellandrene	MT	1044	91;93;79;77	34.44 ± 3.29 ^f,g,A^	14.18 ± 1.22 ^e,C^	26.70 ± 2.57 ^c,d,e,B^	12.74 ± 1.56 ^c,d,e,C^
γ-Terpinene	MT	1047	67;95;108;193	28.72 ± 2.14 ^f,g,A^	1.88 ± 0.09 ^e,C^	30.35 ± 3.04 ^c,d,e,B^	15.22 ± 1.68 ^c,d,e,A^
Thymol methyl ether	AOMT	1057	149;91;164;117	202.55 ± 15.42 ^d,A^	171.39 ± 16.12 ^c,A^	52.56 ± 7.88 ^c,d,e,C^	125.19 ± 11.42 ^c,d,B^
Caryophyllene	SQT	1063	91;105;133;77	193.94 ± 14.69 ^d,e,A^	189.42 ± 1.56 ^c,A^	145.30 ± 15.22 ^c,B^	125.33 ± 14.18 ^c,d,B^
δ-Cadinene	SQT	1073	93;95;91;121	9.69 ± 0.87 ^g,A^	5.33 ± 0.49 ^e,B^	10.99 ± 1.25 ^d,e,A^	4.25 ± 1.77 ^e,B^
γ-Muurolene	SQT	1078	161;105;91;204	88.60 ± 8.36 ^e,f,g,A^	59.88 ± 5.74 ^d,e,A^	96.86 ± 44.12 ^c,d,e,A^	50.19 ± 4.12 ^c,d,e,A^
Bicyclogermacrene	SQT	1089	91;105;133;189	41.64 ± 4.25 ^f,g,A^	30.97 ± 2.09 ^d,e,B^	8.35 ± 0.92 ^d,e,D^	16.48 ± 1.99 ^c,d,e,C^
γ-Cadinene	SQT	1092	161;105;91;119	24.75 ± 2.21 ^f,g,B^	19.65 ± 1.74 ^e,B^	43.03 ± 3.39 ^c,d,e,A^	25.63 ± 2.12 ^c,d,e,B^
α-Calacorene	SQT	1124	91;93;67;79;121	6.27 ± 0.52 ^g,A^	5.18 ± 0.49 ^e,A^	5.53 ± 0.55 ^d,e,A^	5.60 ± 1.13 ^d,e,A^
Caryophyllene oxide	OSQT	1243	429;355;430;295	41.04 ± 3.22 ^f,g,A^	8.25 ± 0.72 ^e,B^	4.76 ± 1.12 ^d,e,B^	10.06 ± 1.31 ^d,e,B^
α-Guaiene	SQT	1255	185;200;201;204	5.17 ± 0.48 ^g,B^	4.66 ± 0.38 ^e,B^	10.82 ± 1.22 ^d,e,A^	6.16 ± 0.74 ^e,B^
γ-Guaiene	SQT	1263	105;133;148;91	17.15 ± 1.62 ^g,A^	12.94 ± 1.16 ^e,A^	17.20 ± 1.97 ^c,d,e,A^	17.16 ± 1.98 ^c,d,e,A^
α-Maaliene	SQT	1270	221;213;429;187	11.83 ± 1.05 ^g,A,B^	8.66 ± 0.71 ^e,B^	12.95 ± 1.42 ^c,d,e,A^	11.22 ± 1.64 ^d,e,A,B^
Thymol	AOMT	1391	135;150;91;115	1674.78 ± 112.88 ^a,A^	1640.55 ± 154.49 ^a,A^	1815.69 ± 200.28 ^a,A^	1611.06 ± 180.14 ^a,A^
Carvacrol	AOMT	1396	135;150;91;115	128.45 ± 13.49 ^d,e,f,A^	124.47 ± 11.76 ^c,d,A^	136.06 ± 14.12 ^c,d,A^	127.65 ± 13.14 ^c,A^
γ-Himachalene	SQT	1398	161;91;135;105	2.68 ± 0.19 ^g,B^	2.41 ± 0.12 ^e,B^	4.24 ± 0.51 ^d,e,A^	3.08 ± 0.28 ^e,B^

* different letters indicate significant differences (*p* < 0.05) among columns (small caps) and rows (capital letters) by post-hoc Tuckey test; MT—monoterpenes; SQT—sesquiterpenes; AOMT—aromatic monoterpenes; OSQT—oxide sesquiterpenes.

## Supplementary Materials

The following are available online at https://www.mdpi.com/2304-8158/9/7/855/s1. Figure S1: Volatile fingerprint of TT-WPC-EF in the beginning of storage, Figure S2: Volatile fingerprint of HPT-WPC-EF in the beginning of storage, Figure S3: Volatile fingerprint of TT-WPC-EF after 10 days of storage, Figure S4: Volatile fingerprint of HPT-WPC-EF after 10 days of storage.
