# Supplementary material for: Thyme Antimicrobial Effect in Edible Films with High Pressure Thermally Treated Whey Protein Concentrate
Source: Foods. 2020 Jun 30;9(7):855. doi: 10.3390/foods9070855 (PMC7404695; doi:10.3390/foods9070855)
Supplement: Supplementary file 1 [file foods-09-00855-s001.pdf]

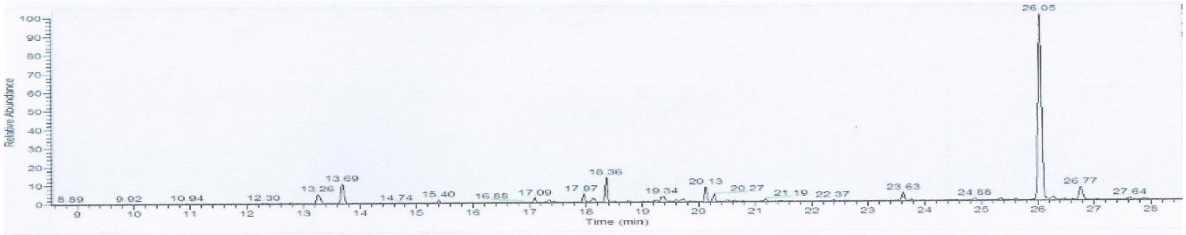

Figure S1. Volatile fingerprint of TT-WPC-EF in the beginning of storage.

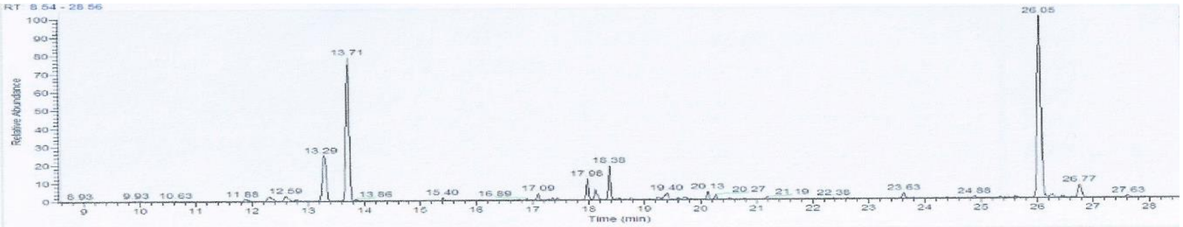

Figure S2. Volatile fingerprint of HPT-WPC-EF in the beginning of storage

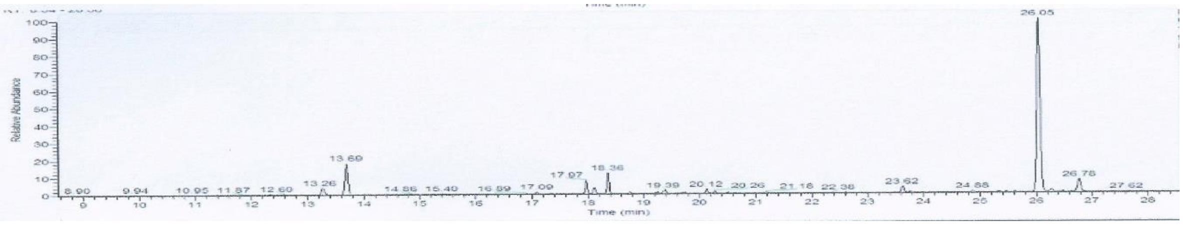

Figure S3. Volatile fingerprint of TT-WPC-EF after 10 days of storage

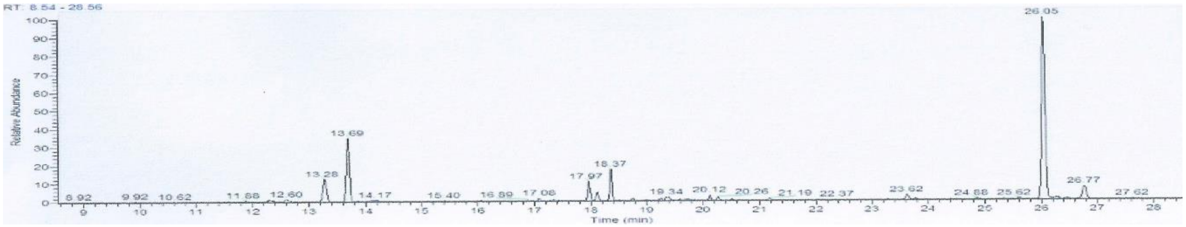

Figure S4. Volatile fingerprint of HPT-WPC-EF after 10 days of storage
